# A trichotomy method for defining homogeneous subgroups in a dementia population

**DOI:** 10.1002/acn3.51869

**Published:** 2023-08-21

**Authors:** Arvind Caprihan, Laura Hillmer, Erik Barry Erhardt, John C. Adair, Janice E. Knoefel, Jillian Prestopnik, Gary A. Rosenberg, for Alzheimer's Disease Neuroimaging Initiative

**Affiliations:** ^1^ The Mind Research Network Albuquerque New Mexico 87106 USA; ^2^ Center for Memory and Aging University of New Mexico School of Medicine Albuquerque New Mexico 87106 USA; ^3^ Departments of Mathematics and Statistics University of New Mexico College of Arts and Sciences Albuquerque New Mexico 87106 USA; ^4^ Department of Neurology University of New Mexico Albuquerque New Mexico 87106 USA

## Abstract

**Introduction:**

Diagnosis of dementia in the aging brain is confounded by the presence of multiple pathologies. Mixed dementia (MX), a combination of Alzheimer's disease (AD) proteins with vascular disease (VD), is frequently found at autopsy, and has been difficult to diagnose during life. This report develops a method for separating the MX group and defining preclinical AD (presence of AD factors with normal cognition) and preclinical VD subgroups (presence of white matter damage with normal cognition).

**Methods:**

Clustering was based on three diagnostic axes: (1) AD factor (ADF) derived from cerebrospinal fluid proteins (Aβ42 and pTau), (2) VD factor (VDF) calculated from mean free water and peak width of skeletonized mean diffusivity in the white matter, and (3) Cognition (Cog) based on memory and executive function. The trichotomy method was applied to an Alzheimer's Disease Neuroimaging Initiative cohort (*N* = 538).

**Results:**

Eight biologically defined subgroups were identified which included the MX group with both high ADF and VDF (9.3%) and a preclinical VD group (3.9%), and a preclinical AD group (13.6%). Cog is significantly associated with both ADF and VDF, and the partial‐correlation remains significant even when the effect of the other variable is removed (*r*(Cog, ADF/VDF removed) = 0.46, *p* < 10^−28^ and *r*(Cog, VDF/ADF removed) = 0.24, *p* < 10^−7^).

**Discussion:**

The trichotomy method creates eight biologically characterized patient groups, which includes MX, preclinical AD, and preclinical VD subgroups. Further longitudinal studies are needed to determine the utility of the 3‐way clustering method with multimodal biological biomarkers.

## Introduction

In 2015 more than 45 million people suffered from Alzheimer's disease and related dementia (ADRD), and this number is expected to dramatically increase to 75 million by 2030, and further to 132 million by 2050.^1^, ^2^ Multiple pathological processes contribute to cognitive impairment in the aging brain and overlapping syndromes confound clinical diagnosis, which can be improved with biomarkers from imaging, cerebrospinal fluid (CSF) and blood. Pathological studies indicate the combination of AD and vascular disease, referred to as mixed dementia (MX), is the most common form of dementia.^3^, ^4^, ^5^ However, diagnosis of MX during life has been problematic, leading to an intense search for biomarkers to improve classification.^6^, ^7^, ^8^, ^9^

Clinical trials that include homogeneous patient groups targeting a specific pathophysiological process are more likely to succeed with fewer participants for adequate statistical power.^10^ The formula, amyloid (A), tau (T), and neurodegeneration (N), improves diagnosis of AD.^11^ While using this formula has improved the diagnosis of AD, it fails to capture several other contributory factors (e.g., vascular disease and synucleinopathy). To remedy that deficiency, an expanded formula that includes other diagnoses, ATXN, has been proposed, where “X” represents novel candidate biomarkers for additional pathophysiological mechanisms.^12^ Adding biomarkers from CSF and positron emission tomography (PET) improves biological diagnosis, advancing AD research, it is magnetic resonance imaging (MRI), especially diffusion imaging that identifies white matter damage, making it a surrogate for vascular damage, but they can be caused by other pathologies as well.^13^ The presence of WMH does not confirm presence of cerebral small vessel disease (CSVD). At present, neuropathology of postmortem brains is the only sure confirmation for the presence of vascular disease, and large differences exist between white matter damage seen in MRI images and CSVD seen in neuropathology.^14^ We know that white matter damage is not unique to CSVD, hence studies that combine MRI‐based markers of white matter damage with fluid biomarkers to give a CSVD diagnosis would be useful. The recent STRIVE‐2 criteria have endorsed both mean free water (mFW) in white matter^15^, ^16^, ^17^ and peak width of skeletonized mean diffusivity (PSMD)^18^ calculated from diffusion tensor imaging, as among the best currently available indicators of microvascular damage to the white matter.^19^ We combined AD proteins in CSF with MRI markers to show white matter injury in a double‐dichotomy clustering classification method that made the diagnosis of MX possible during life and separated dementia patients into four groups.^20^ However, our prior report had several drawbacks, including the small sample size from a single center and lack of a cognitive dimension. In this report we expanded the number of subjects to over 500 with the Alzheimer's Disease Neuroimaging Initiative (ADNI) database and added a cognitive dimension to the double‐dichotomy, forming a three‐dimensional “trichotomy” clustering method that improved homogeneity by creating eight patient groups.

The ATN framework was based on each axis being defined by the three different pathological processes appropriate for AD diagnosis.^11^ Our proposed system uses three‐axes based on the goal of distinguishing Alzheimer's and vascular disease, and to further distinguish subjects with normal and low cognitive performance. Our proposal combines the “AT” of ATN into one Alzheimer's disease factor (ADF) and adds two other independent measures to characterize vascular disease, a vascular disease factor (VDF) and a composite cognition (Cog) measure of executive and memory function. They are defined as functions of appropriate biomarkers with their range in [0,1] and a cut‐point = 0.5. The three scores are plotted orthogonally and the threshold of 0.5 divides the subjects into eight groups.

## Materials and Methods

### Subjects

The participants were selected from the Alzheimer's Disease Neuroimaging Initiative (ADNI2 and ADNI3) database (http://adni.loni.usc.edu) if they had, (a) CSF measurements of Aβ42 and pTau, (b) MRI diffusion and FLAIR measurements, and (c) composite scores for memory (ADNI_MEM) and executive function (ADNI_EF). The ADNI was initiated in 2003 by NIH under the leadership of Dr. Weiner. The primary objectives of ADNI were to identify biomarkers to measure progression of mild cognitive impairment (MCI) and early identification of AD (www.adni‐info.org). Institutional review board approval was obtained from each of the multicenter sites, and an informed consent was obtained for each study participant. We maintained the original ADNI diagnostic classification for reference with a change in the naming convention to distinguish the original clinical diagnosis from our proposed biological diagnosis. ADNI classifies the subjects into three groups based on a clinical and neuropsychological evaluation: (a) cognitively normal (aCN), (b) mild cognitive impairment (aMCI), and (c) Alzheimer's disease (aAD). The subject demographics are in Table 1.

**Table 1:** Demographics of participants. aCN, aMCI, aAD are the three groups defined in the ADNI database as cognitively normal, mild cognitive impairment, and Alzheimer's disease.

Measure	aCN	aMCI	aAD	Total
Participants	283	186	69	538
Age	71.2 (6.3) [66.85, 75.45]	71.9 (7.5) [66.8, 77.48]	72.8 (8.2) [66.1, 77.7]	71.7 (7.0) [66.8, 75.45]
Sex (males %)	39.9%	57.0%	60.9%	48.5%
Education	16.8 (2.3) [16, 18]	16.1 (2.6) [14, 18]	15.6 (2.6) [14, 18]	16.4 (2.5) [14.25, 18]
12 < education ≤ 16	41.0%	47.3%	52.2%	44.7%
Education >16	52.9%	39.2%	30.4%	44.8%
Ethnicity: Not Hispanic or Latino	93.6%	95.7%	89.9%	93.9%
Race: White	91.5%	90.9%	98.6%	92.2%
BMI	27.4 (5.0) [23.8, 30.2]	27.6 (4.7) [24.5, 29.8]	26.6 (5.4) [23.0, 28.6]	27.4 (5.0) [24.0, 29.9]
BP Systole	135.1 (16.2) [125.0, 145.0]	134.4 (16.8) [123.0, 145.0]	134.8 (16.7) [123.0, 145.0]	134.8 (16.4) [24, 145.5]
Hypertension (exists)	86.2%	89.8%	92.8%	88.3%
High cholesterol (exists)	54.1%	61.3%	59.4%	57.2%
Diabetes (exists)	12.4%	14.0%	13.0%	13%

Summaries are mean (SD) [25%, 75%] for numeric values, and percent for categorical values.

The eight biologically defined groups are summarized in Table 2. In the earlier work^20^ the bCN group (cognitively normal with no AD or VD factors) was diagnosed separately and was not part of the double‐dichotomy analysis. The four groups shared with the double‐dichotomy methods are, bMX = biological mixed dementia, bAD = biological Alzheimer's disease, bVD = biological vascular disease, and bCN_VD_ = biological leukoaraiosis (normal cognition with white matter changes on FLAIR). The three new groups are, bCL = cognitively low performers with no AD or VD factors, bCN_MX_ = cognitively normal with both AD and VD factors, and bCN_AD_ = cognitively normal with only AD factors. The cognitively normal groups with AD or VD factors may be important in a longitudinal study of disease progression.

**Table 2:** The classification criteria and the nomenclature of the eight biologically defined subgroups is described below.

Trichotomy subgroups	Trichotomy notation	Cognition cog	Alzheimer's disease factor ADF	Vascular disease factor VDF
Biological mixed dementia	bMX	+	+	+
Biological Alzheimer's disease	bAD	+	+	−
Biological vascular disease SIVD (subcortical ischemic vascular disease)	bVD	+	−	+
Cognitively low performers with no AD or VD factors	bCL	+	−	−
Cognitively normal with AD and VD factors Preclinical MX	bCN_MX_	−	+	+
Cognitively normal with AD factors Preclinical AD	bCN_AD_	−	+	−
Cognitively normal with VD factors Preclinical VD	bCN_VD_	−	−	+
Cognitively normal with no AD or VD factors	bCN	−	−	−

The groups closest to clinically accepted definitions of subcortical ischemic vascular disease (SIVD) is indicated in the table. The definition of preclinical AD is the presence of AD factors but being cognitively normal, and similarly preclinical VD is the presence of white matter damage due to normal aging with no cognitive decline.

### Biochemical assessments

ADNI used the Elecsys system for measuring Aβ42 (pg/mL) and pTau (pg/mL) values in CSF. We obtained these values from the ADNI database. Several previous studies have determined suitable cutoff values by comparing these CSF‐based measurements to ^18^F PET studies.^21^, ^22^ There are other studies that have shown that using ratios (Aβ42/Aβ40, pTau/Aβ42, pTau/Aβ40) is better than using individual biomarkers for predicting clinical progression of AD or for predicting ^18^F PET status.^21^, ^23^, ^24^ This study uses a composite score based on the ratio pTau/Aβ42 to characterize AD. A larger value of the composite score captures the low concentration of Aβ42 and the high concentration of pTau present in AD. A cutoff value of 0.022 was used to define AD‐positive subjects.^21^

### Composite cognitive scores

The methods of obtaining composite memory function and executive function scores and the advantages of using composite scores have been previously summarized.^25^, ^26^, ^27^ ADNI_MEM was based on longitudinal Rey Auditory Verbal Learning Test (RAVLT, 2 version), AD Assessment Schedule—Cognition (ADAS‐Cog, 3 version), Mini‐Mental State Examination (MMSE), and Logical Memory Data. ADNI_EF was based on WAIS‐R Digit Symbol Substitution, Digit Span Backwards, Trails A and B, Category Fluency, and Clock Drawing.

### MRI acquisition

The MRI protocol details are available (https://adni.loni.usc.edu/). The ADNI2 dataset has thick slices for the FLAIR sequence and larger voxel size for the diffusion sequence. All diffusion calculations were only done with shells *b* = 1000 s/mm^2^ and *b* = 0 s/mm^2^, with higher order shells excluded (ADNI3 advanced protocol). This minimized the difference between diffusion measures calculated from different ADNI datasets. The white matter hyperintensity volume (WMHV), mFW in white matter,^15^ and PSMD^18^ were calculated based on methods described earlier,^28^ and the scripts available on the MarkVCID website (https://markvcid.partners.org/consortium‐protocols‐resources). In all regression analyses a protocol variable was used to account for differences across ADNI protocols.

### Calculation of normalized composite scores

**Figure 1:**
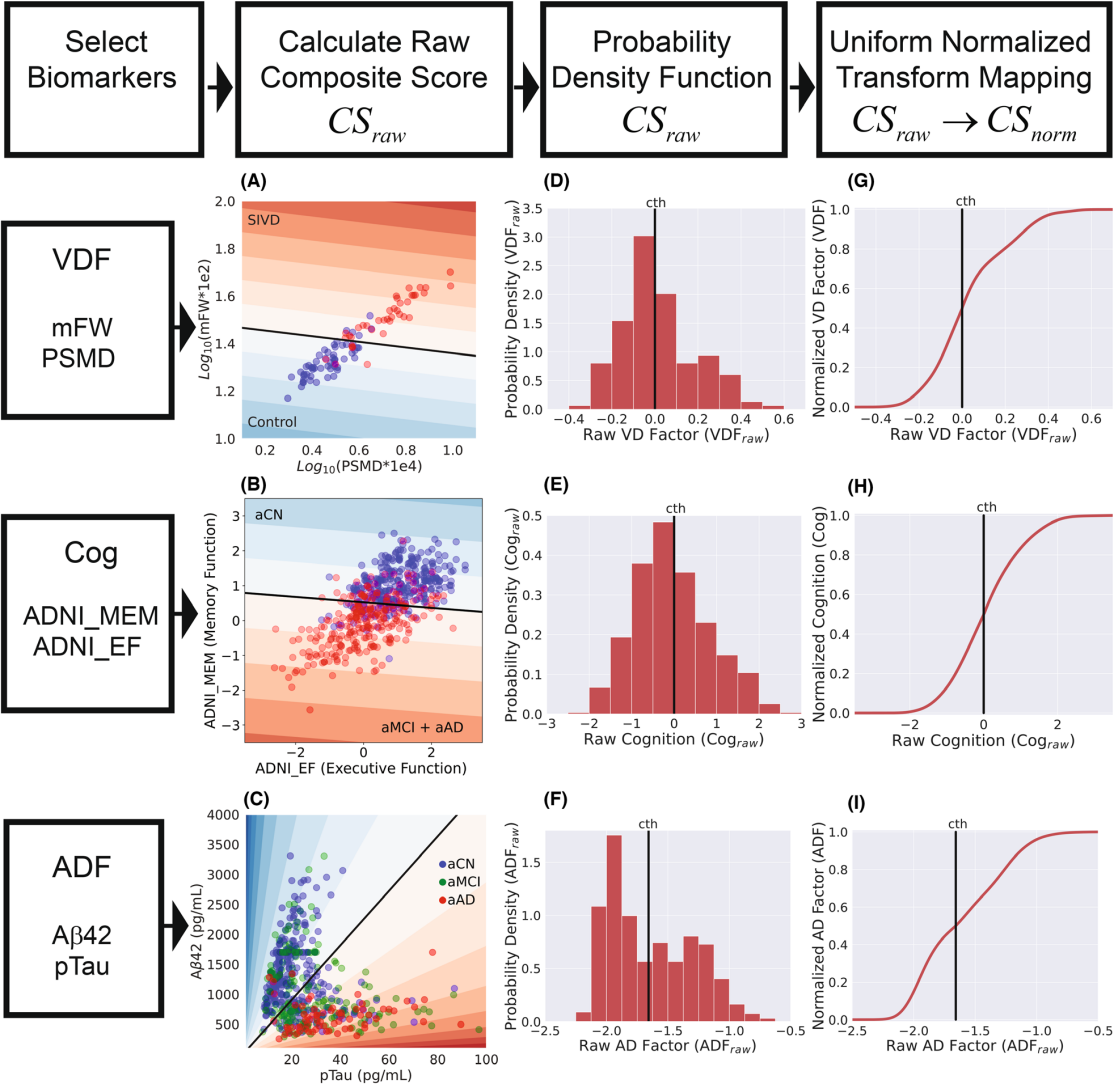
The top row summarizes the four steps required for calculating a normalized composite score. The next three rows show details of the steps required for calculating the three different composite scores: (1) Vascular disease factor (VDF), (2) Cognition (Cog), and (3) Alzheimer's disease factor (ADF). The black line in (A–C) defines the cut‐off for the three examples, with the colors being the contour plots of the raw composite score. (A) is the contour diagram of the raw score, ${\mathrm{VDF}}_{\mathrm{raw}}=0.135{\mathrm{Log}}_{10\times \times }\left(\text{PSMD×}1e^{4}\right)+0.991{\mathrm{Log}}_{10}\left(\mathrm{mFW}\times 1e^{2}\right)-1.474$, with the black line being ${\mathrm{VDF}}_{\mathrm{raw}}=0$, and contour lines being ${\mathrm{VDF}}_{\mathrm{raw}}=0.1$ apart, with red being the positive score. (B) is the contour diagram of the raw score, ${\mathrm{Cog}}_{\mathrm{raw}}=-0.076\text{ADNI}\_\mathrm{EF}-0.997\text{ADNI}\_\mathrm{MEM}+0.519$, with the black line being ${\mathrm{Cog}}_{\mathrm{raw}}=0$, and contour lines being ${\mathrm{Cog}}_{\mathrm{raw}}=1$ apart, with red being the negative score. (C) is the contour diagram of the raw score, ${\mathrm{ADF}}_{\mathrm{raw}}={\mathrm{Log}}_{10}\left(\text{pTau}/\mathrm{A\beta}42\right)$, with the black line being ${\mathrm{ADF}}_{\mathrm{raw}}=Log_{10}\left(0.022\right)$, and contour lines being ${\mathrm{ADF}}_{\mathrm{raw}}=0.2$ apart, with red being the positive score. The contour lines are at an angle because ${\mathrm{ADF}}_{\mathrm{raw}}$ is the ratio of the two variables.

The four steps for calculating ${\mathrm{CS}}_{\mathrm{norm}}$ are summarized in Figure 1 and the mathematical details are described in the Supplementary Materials. We (1) select the biomarkers for defining the composite score, (2) define the mathematical formula for calculating the raw composite score (${\mathrm{CS}}_{\mathrm{raw}}$) and a cutoff (cth) based on a classification algorithm (for ${\mathrm{VDF}}_{\mathrm{raw}}$ and ${\mathrm{Cog}}_{\mathrm{raw}}$) or an independent study (for ${\mathrm{ADF}}_{\mathrm{raw}}$), (3) calculate $f\left({\mathrm{CS}}_{\mathrm{raw}}\right)$, the probability density function of ${\mathrm{CS}}_{\mathrm{raw}}$, and (4) calculate a uniform normalization transform (UNT, a function $f\left({\mathrm{CS}}_{\mathrm{raw}}\right)$) to map ${\mathrm{CS}}_{\mathrm{raw}}$ to ${\mathrm{CS}}_{\mathrm{norm}}$ in the range (0,1) and the cutoff to 0.5. ${\mathrm{CS}}_{\mathrm{norm}}$ is a monotonically increasing function of ${\mathrm{CS}}_{\mathrm{raw}}$, which maintains the relative order of individual scores, and ${\mathrm{CS}}_{\mathrm{norm}}$ is approximately uniformly distributed in the range (0,1). The method to calculate ${\mathrm{CS}}_{\mathrm{raw}}$ was slightly different for the three biomarkers (VDF, Cog, and ADF), while the same algorithm transformed ${\mathrm{CS}}_{\mathrm{raw}}$ to ${\mathrm{CS}}_{\mathrm{norm}}$ for all three biomarkers. The method for calculating ${\mathrm{CS}}_{\mathrm{raw}}$ and the details of mapping ${\mathrm{CS}}_{\mathrm{raw}}$ to ${\mathrm{CS}}_{\mathrm{norm}}$ are described with greater detail in the Supplemenatry materials.

${\mathrm{VDF}}_{\mathrm{raw}}$ was calculated as a linear function of white matter mFW and PSMD calculated from a diffusion image. The linear function was defined by linear discriminant analysis (LDA) of separating the UNM cohort into controls and subcortical ischemic vascular disease (SIVD) groups (Fig. 1A). The UNM data were used for calculating ${\mathrm{VDF}}_{\mathrm{raw}}$ because they had recruited subjects with clinically diagnosed vascular cognitive impairment and included a more extensive range of white matter damage than the ADNI dataset. White matter hyperintensity volume (WMHV) was excluded for calculating the composite scores because it did not improve the classification of subjects into control and patient groups, both mFW and PSMD had higher correlation with executive function than WMHV, and they do not depend on a binary threshold to detect white matter lesions and can continuously monitor white matter changes. The classification accuracy of the LDA algorithm for separating the controls from the SIVD group in the UNM cohort was 88.3% (Supplementary Materials for further details). ${\mathrm{VDF}}_{\mathrm{raw}}$ is given by,

(1) $$\begin{array}{l} {\mathrm{VDF}}_{\mathrm{raw}}=0.135{\mathrm{Log}}_{10}\left(\text{PSMD}\times 1e^{4}\right)\\ \;+\;0.991{\mathrm{Log}}_{10}\left(\mathrm{mFW}\times 1e^{2}\right)-1.474,\\ \text{with}\;\mathrm{cth}=0. \end{array}$$

${\mathrm{Cog}}_{\mathrm{raw}}$ was calculated was calculated as a linear function of ADNI_MEM and ADNI_EF. The ADNI data (538 subjects) were used to define a linear discriminant function that best separated subjects with normal cognition (aCN) and those with some cognitive impairment (aMCI + aAD). An independent dataset was not available in this case for calculating ${\mathrm{Cog}}_{\mathrm{raw}}$. The previous LDA method was used, and additionally we validated the stability of the ${\mathrm{Cog}}_{\mathrm{raw}}$ by leave‐one‐out cross‐validation method (Fig. 1B). ${\mathrm{Cog}}_{\mathrm{raw}}$ is given by,

(2) $$\begin{array}{l} {\mathrm{Cog}}_{\mathrm{raw}}=-0.076\text{ADNI}\_\mathrm{EF}-0.997\text{ADNI}\_\mathrm{MEM}+0.519,\\ \text{with}\;\mathrm{cth}=0. \end{array}$$

This classification rule had 82.2% accuracy for separating the ADNI group aCN from the combined aMCI + aAD groups.

${\mathrm{ADF}}_{\mathrm{raw}}$ was based on the results of an independent study, which used the ratio of Aβ42 to pTau and calculated a cutoff that best matched the PET results for AD presence^21^ (Fig. 1C). ${\mathrm{ADF}}_{\mathrm{raw}}$ is given by,

(3) $$\begin{array}{l} {\mathrm{ADF}}_{\mathrm{raw}}={\mathrm{Log}}_{10}\left(\text{pTau}/\mathrm{A\beta}42\right),\\ \text{with}\;\mathrm{cth}={\mathrm{Log}}_{10}\left(0.022\right)=-1.66. \end{array}$$

All the three raw scores, ${\mathrm{VDF}}_{\mathrm{raw}}$, ${\mathrm{Cog}}_{\mathrm{raw}}$ and ${\mathrm{ADF}}_{\mathrm{raw}}$ were converted to normalized scores ${\mathrm{VDF}}_{\mathrm{norm}}$, ${\mathrm{Cog}}_{\mathrm{norm}}$, and ${\mathrm{ADF}}_{\mathrm{norm}}$ by the table based UNT method described in Supplementary Materials (Fig. 1G–I).

## Results

### Relationships between Biomarkers

Table 3 summarizes the linear regression results of the relationships between the biomarkers with age, sex, education, protocol differences, and an additional variable as indicated, treated as a covariate. The partial correlation was calculated between the residuals after removing the effect of covariates from each variable.

**Table 3:** Results of linear regression and partial correlation after taking into effects of age, sex, education, and an additional covariate if present are shown.

Row	Biomarker1	Biomarker2	Additional covariate	Beta	SE	Partial correlation	*p*‐value
1	ADNI_MEM	ADNI_EF		0.48	0.029	0.6	<10^−52^
2	Aβ42	pTau		−0.23	0.05	−0.21	<10^−5^
3	mFW	PSMD		0.41	0.018	0.72	<10^−75^
4	VDF	Aβ42		−0.15	0.03	0.2	<10^−2^
5	VDF	pTau		0.01	0.04	0.1	ns
6	ADF	mFW		0.56	0.17	0.21	<10^−3^
7	ADF	PSMD		0.19	0.1	0.13	ns
8	Cog	VDF		0.37	0.057	0.31	<10^−9^
9	Cog	ADF		0.44	0.035	0.49	<10^−30^
10	ADNI_MEM	ADF		−1.35	0.11	−0.49	<10^−30^
11	ADNI_EF	ADF		−1.26	0.14	−0.39	<10^−17^
12	ADNI_MEM	VDF		−1.1	0.17	−0.3	<10^−9^
13	ADNI_EF	VDF		−1.6	0.21	−0.38	<10^−13^
14	VDF	ADF		0.09	0.03	0.2	<10^−2^
15	Cog	VDF	ADF	0.29	0.051	0.24	<10^−7^
16	Cog	ADF	VDF	0.42	0.035	0.46	<10^−28^
17	ADNI_MEM	ADF	VDF	−1.27	0.11	−0.46	<10^−28^
18	ADNI_EF	ADF	VDF	−1.13	0.14	−0.34	<10^−15^
19	ADNI_MEM	VDF	ADF	−0.85	0.16	−0.23	<10^−7^
20	ADNI_EF	VDF	ADF	−1.4	0.2	−0.29	<10^−11^

Partial correlation is calculated from the residuals after removing the effects of the covariates from Biomarker1 and Biomarker2.

Biomarker pairs within each modality ([ADNI_EF and ADNI_MEM], [mFW and PSMD] and [Aβ42 and pTau]) are significantly correlated with each other (Rows 1–3). The association between Aβ42 and pTau was the weakest among the three pairs.

The relationship of the individual biomarkers across modalities was different. VDF was not significantly associated with pTau, while it was significantly associated with Aβ42 (Rows 4–5). Similarly, ADF was not significantly associated with PSMD, while it was associated with mFW (Rows 6–7). Although the pair pTau and Aβ42, and the pair mFW and PSMD are correlated with each other, they do contribute with independent information in defining the composite measures.

The composite (Cog) and the individual cognitive measures (ADNI_MEM, ADNI_EF) are significantly associated with both ADF and VDF, with ADF having a higher correlation then VDF (Rows 8–13). Although ADF and VDF are significantly correlated with each other (Row 14), the partial correlation between the cognitive measures and VDF remains significant, even after the effects of ADF are removed (for example, *r*(Cog, VDF/ADF removed) = 0.29 *p* < 10^−7^), and similarly the partial correlation between the cognitive measures and ADF remains significant after the effects of VDF are removed (for example, *r*(Cog, ADF/VDF removed) = 0.42), *p* < 10^−28^) (Rows 15–20). ADF had higher correlation with memory than with the executive function (ADNI_EF) (Rows 10–11 and 17–18), while VDF had higher correlation with the executive function than with memory (Rows 12–13, and 19–20).

The variables mFW and PSMD are highly correlated, and we examine their relative value in predicting Cog. Table 4 lists *R* ^2^ and Akaike Information criteria (AIC) for the model, predicting Cog based on ${\mathrm{Log}}_{10}\left(\mathrm{mFW}\right)$, and ${\mathrm{Log}}_{10}\left(\text{PSMD}\right)$. The value of *R* ^2^ always decreases with increased number of variables, but a lower value of AIC indicates a parsimonious model‐fit, because it penalizes increased number of variables. There is a slight increase in the explained variance if both the variables, mFW and PSMD are included for predicting Cognition. The AIC suggest that the increased complexity of adding PSMD is not justified in a model to predict Cog based on ${\mathrm{Log}}_{10}\left(\mathrm{mFW}\right)$ and on ${\mathrm{Log}}_{10}\left(\text{PSMD}\right)$. This calculation shows that in subsequent versions of the method, mFW should be sufficient. This conjecture must be examined over other datasets which include subjects with increased amounts of vascular disease.

**Table 4:** The relative importance of mFW and PSMD in predicting cognition is compared.

Regression model	*R* ^2^	AIC
Cog ~ ${\mathrm{Log}}_{10}\left(\text{PSMD}\right)$	0.11	42.29
Cog ~ ${\mathrm{Log}}_{10}\left(\mathrm{mFW}\right)$	0.25	18.45
Cog ~ ${\mathrm{Log}}_{10}\left(\mathrm{mFW}\right)+{\mathrm{Log}}_{10}\left(\text{PSMD}\right)$	0.26	20.39

### Trichotomy‐based subgroups

**Figure 2:**
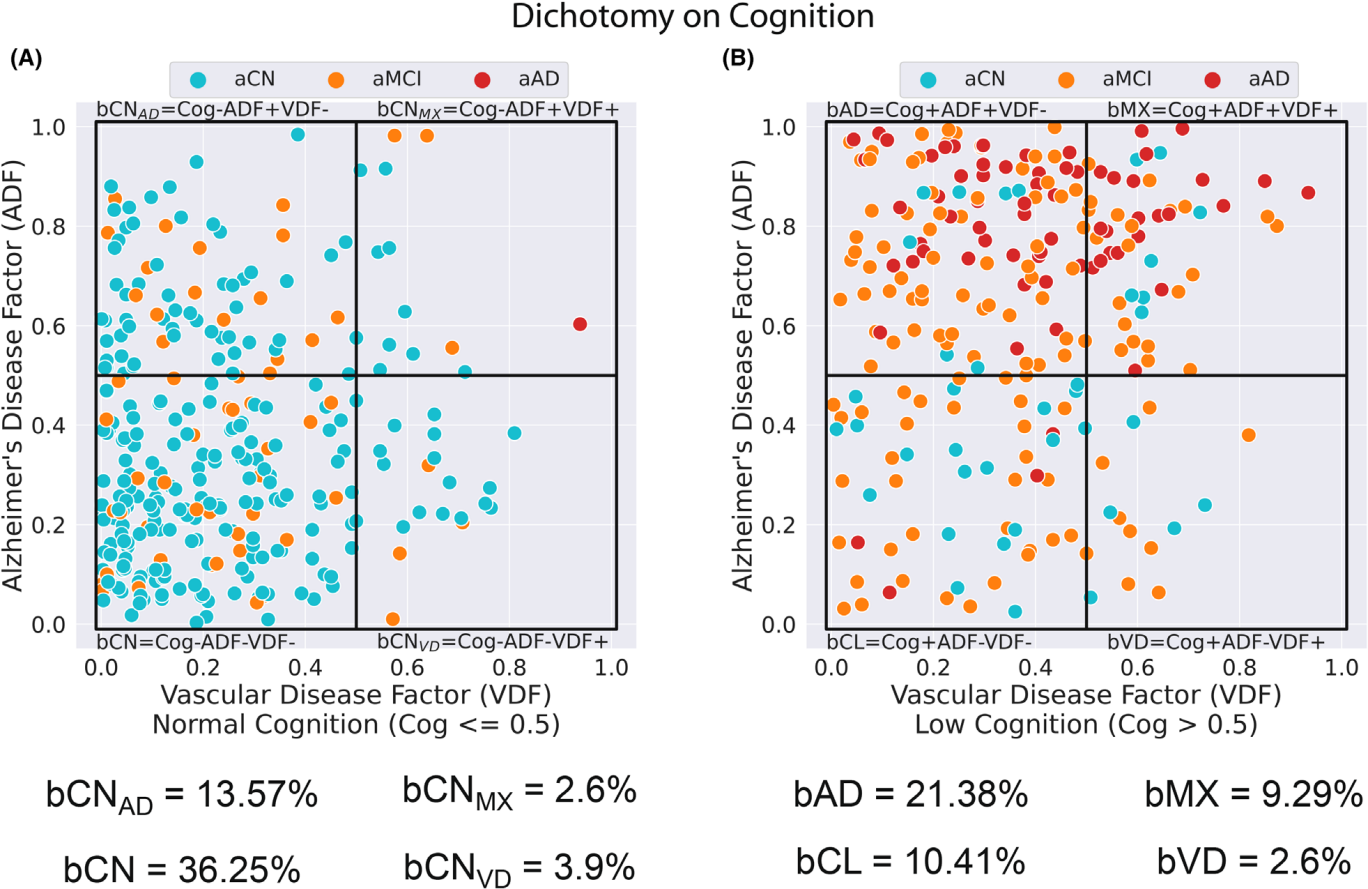
The trichotomy plot (VDF, ADF, Cog) was divided into two figures, based on a dichotomy on cognition, Cog ≤0.5 (A) and Cog >0.5 (B). The colors distinguish the original ADNI classification (aCN, aMCI, and aAD). This figure reflects the characteristics of the ADNI database. Majority of the subjects fall in bCN and bAD groups (57.6%, bCN + bAD) with considerably lower number of subjects have the vascular disease factor (18.4%, bCN_MX_ + bCN_VD_ + bMX + bVD). This analysis identifies subjects with mixed dementia (bMX) and those with preclinical VD (bCN_VD_) and preclinical AD (bCN_AD_).

The trichotomy defined sub‐groups (Table 2), along with the subject distribution across the eight groups is shown in Figure 2. A dichotomy based on cognition, splits the ADNI group into two parts based on Cog <= 0.5 (Fig. 2A) and Cog >0.5 (Fig. 2B). Figure S1 in the Online Appendix shows the six figures based on the dichotomy of Cog, ADF, and VDF, respectively. The numerical distribution of the subjects across our eight biologically defined groups and the ADNI groups (aCN, aMCI, and aAD) is shown in Table S1.

Overall, 9.3% of ADNI subjects were classified as mixed dementia (bMX), while in the Alzheimer's ADNI group (aAD) 22 of 69 subjects (31.9%) had mixed dementia. In 102 of 283 (36.0%) cognitively normal ADNI subjects (aCN) Alzheimer's or vascular disease factors were present. Finally, 107 of 186 (57.5%) of mild cognitively impaired ADNI subjects (aMCI) had AD factors (groups bMX, bAD, bCN_MX,_ and bCN_AD_).

We consider preclinical to be those with normal cognition but having biological factors (bCN_VD_, bCN_AD_, and bCN_MX_). Overall, 3.9% of the subjects were classified as bCN_VD_ (21 out of 538 subjects), 13.5% were classified as bCN_AD_ (73 out of 538 subjects), and 2.6% were classified as bCN_MX_ (14 out or 538 subjects).

### Biomarker properties for the ADNI subgroups

**Figure 3:**
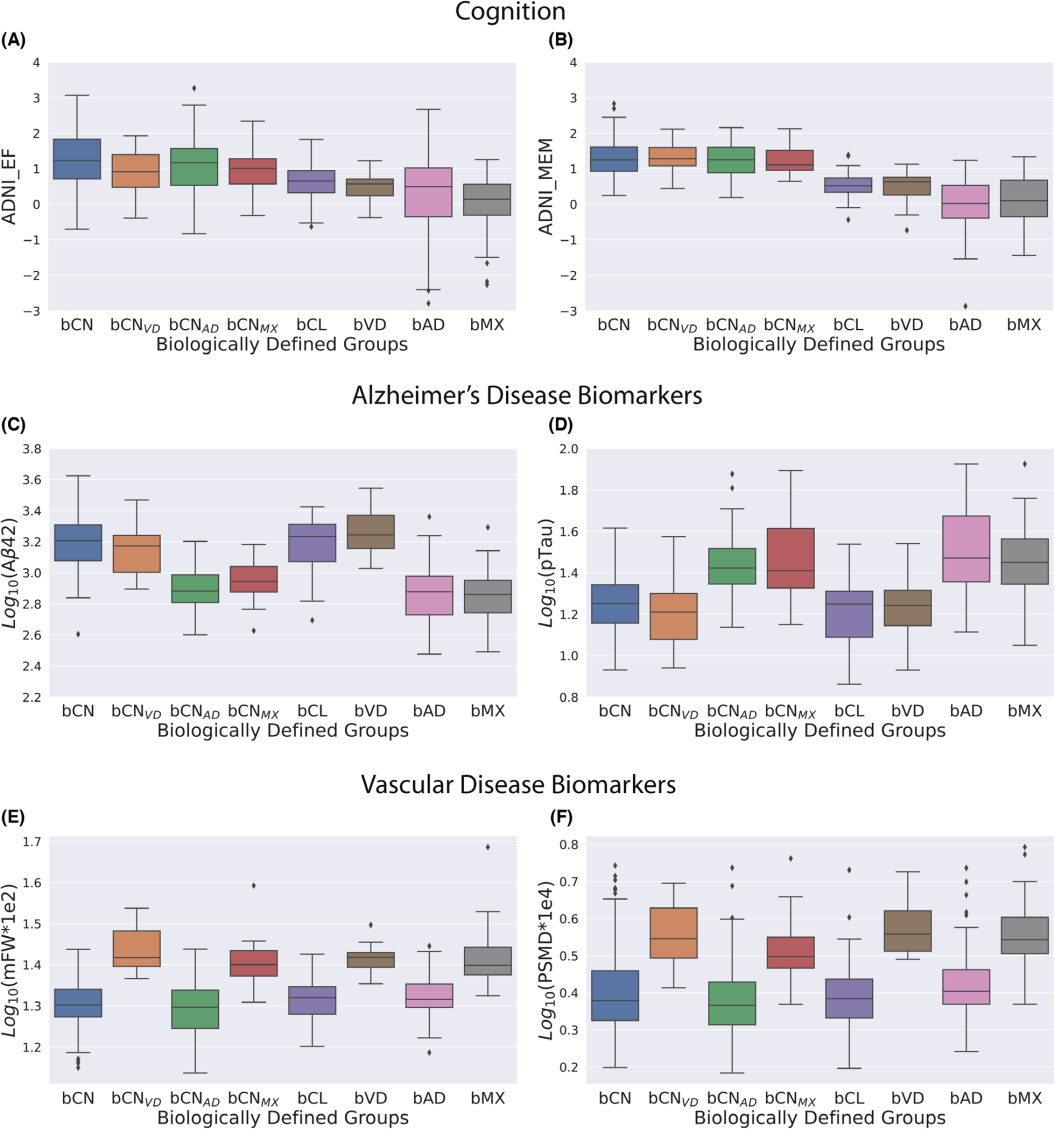
The distribution of the biomarkers that were used for calculating the composite scores is compared across the eight trichotomy groups. The variation of each marker is as expected based on how the groups were constructed. The memory is not affected by AD or VD factors within the cognitively normal groups, but within the cognitively low performance groups (bCL + bVD + bAD + bMX), the memory function is significantly lower in those with AD factors (bAD + bMX) than those without (bCL + bVD).

**Figure 4:**
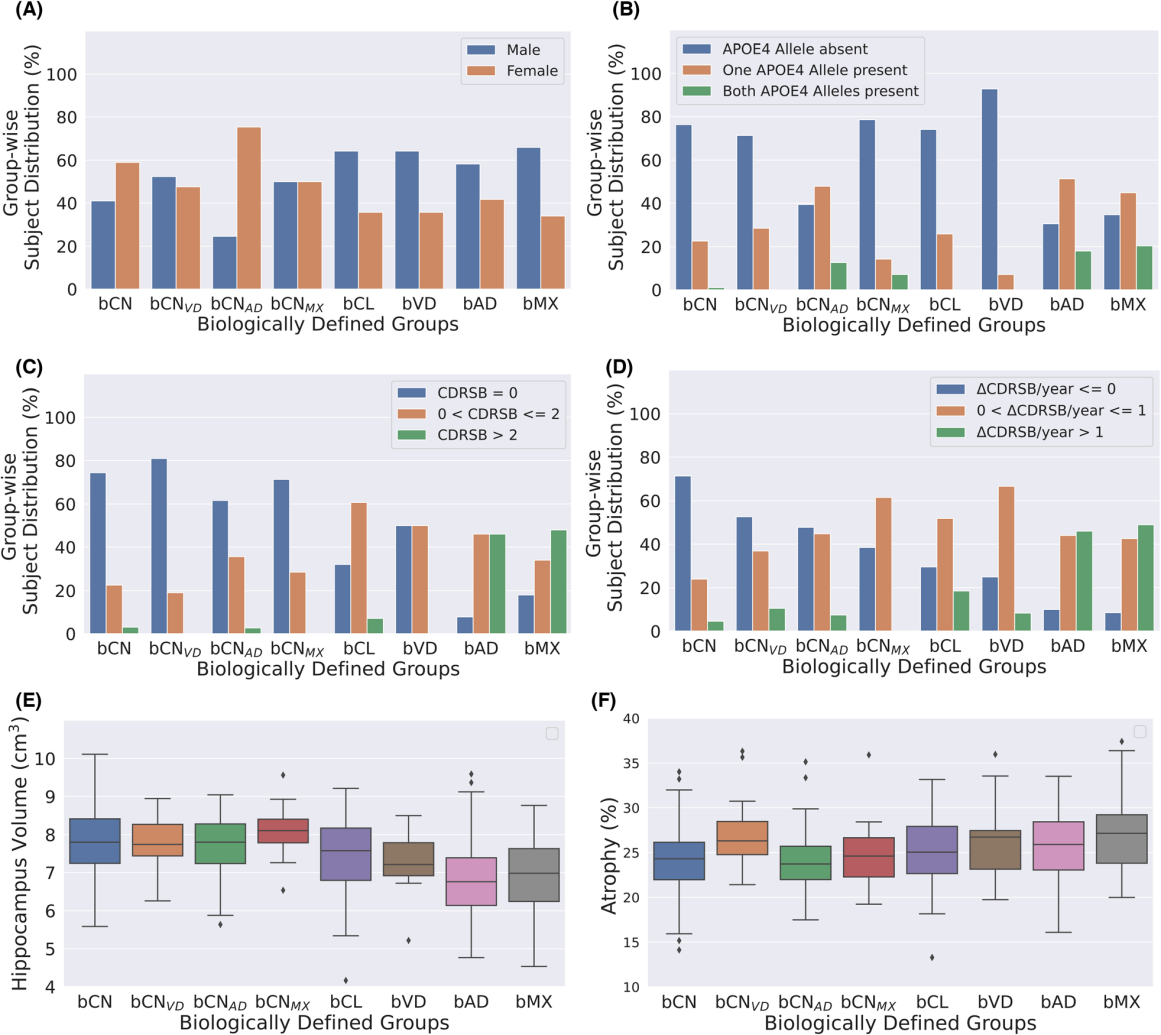
The distribution of the biomarkers that were not used for calculating the composite scores is compared across the eight trichotomy groups. The statistical differences between the groups are discussed in Table 5. There is a greater proportion of males in cognitively low groups with presence of AD. In subjects with AD and low cognition there was a greater proportion of subjects present with (a) both alleles present (Fig. 3B), (b) higher values of CDRSB (Fig. 3C) and (c) higher values of ΔCDRSB/year (Fig. 3D), as seen by the green bars. Hippocampal volume was less in groups with AD and it was more sensitive than brain atrophy to group differences (Fig. 3E,F).

We first discuss the variation of the six biomarkers used in defining the composite scores across the eight groups (Fig. 3). Next, we discuss the difference across the eight groups for six additional biomarkers that were not used in defining the composite scores (Fig. 4).

The differences in cognition, Alzheimer's disease factors, and vascular disease factors across the eight groups are shown in Figure 3. The variation of the biomarkers across the eight groups follows the expected differences based on how the groups were created. In Figure 3F there are subjects in the bAD group with large PSMD values that were not classified as bMX. This occurs because the composite VDF score primarily depends on mFW (Eq. 1), and mFW is small for these subjects. In the groups with normal cognition there was minimal effect of disease on ADNI_EF and none on ADNI_MEM. ADNI_EF (bCN) was significantly greater than ADNI_EF (bCN_AD_ + bCN_MX_ + bCN_VD_) with *p* < 0.02). On the other hand, in the cognitively low performance groups the presence of VDF decreased ADNI_EF, while the presence of ADF decreased ADNI_MEM. ADNI_EF (bCL + bAD) was significantly greater than ADNI_EF (bVD + bMX) with *p* < 0.02, and ADNI_MEM (bCL + bVD) was significantly greater than ADNI_MEM (bAD + bMX) with *p* < 1e‐5.

Figure 4 compares the distribution of the six variables that were not used in defining the composite scores. These being (a) sex, (b) APOE4, (c) CDR sum of boxes (CDRSB) (d) annual change of CDR sum of boxes (ΔCDRSB/year), (e) hippocampus volume (HV), and (f) brain atrophy (BA). Chi‐squared test was used to test for equivalence of proportions in two groups and two‐sample *t*‐test was used to compare means. Except for HV and BA, the other measures are proportion of subjects. The nine pairs of groups that we compare are summarized in Table 5A and the nine hypothesis we test are summarized in Table 5B. Table 5C gives the *p*‐values for testing each hypothesis being different across each pair of groups. The unadjusted *p*‐values were multiplied by 81 for Bonferroni multiple‐comparison correction.

**Table 5:** We test the significance of nine hypothesis based on the distribution of the six variables (Fig. 4) across the eight biological groups. (A) Gives the different combination of groups being compared based on the eight biological groups. (B) Gives the nine hypotheses being tested based on the Figure 4 biomarkers. (C) Gives the multiple‐comparison corrected *p*‐values for testing each hypothesis.

(A)			
	Groups description	Groups being compared	
		Group 1	Group 2
G1	Cognition (low vs. normal)	bCL + bVD + bAD + bMX	bCN + bCN_VD_ + bCN_AD_ + bCN_MX_
G2	Vascular disease (VD) (yes vs. no)	bVD + bMX + bCN_VD_ + bCN_MX_	bCN + bCN_AD_ + bCL + bAD
G3	Alzheimer's disease (AD) (yes vs. no)	bAD + bMX + bCN_AD_ + bCN_MX_	bCN + bCN_VD_ + bCL + bVD
G4	Cognition (low vs. normal) for VD‐Yes	bVD + bMX	bCN_VD_ + bCN_MX_
G5	Cognition (low vs. normal) for AD‐Yes	bAD + bMX	bCN_AD_ + bCN_MX_
G6	VD (yes vs. no) for Cognition – Low	bVD + bMX	bCL + bAD
G7	VD (yes vs. no) for AD‐Yes	bMX + bCN_MX_	bAD + bCN_AD_
G8	AD (yes vs. no) for Cognition‐Low	bAD + bMX	bCL + bVD
G9	AD (yes vs. no) for VD‐Yes	bMX + bCN_MX_	bVD + bCN_VD_

(B)		
	Figure	Hypothesis
H1	Figure 3A	Proportion of males in Group1 > those in Group 2
H2	Figure 3B	Proportion of subjects with at least one allele in Group 1 < those in Group 2
H3	Figure 3B	Proportion of subjects with two alleles in Group 1 > those in Group 2
H4	Figure 3C	Proportion of subjects with (CDRSB >0) in Group 1 > those in Group 2
H5	Figure 3C	Proportion of subjects with (CDRSB >2) in Group 1 < those in Group 2
H6	Figure 3D	Proportion of subjects with (ΔCDRSB/year >0) in Group 1 > those in Group 2
H7	Figure 3D	Proportion of subjects with (ΔCDRSB/year >1) in Group 1 < those in Group 2
H8	Figure 3E	HV mean for Group 1 > that for Group 2
H9	Figure 3F	BA mean for Group 1 > that for Group 2

(C)									
	H1	H2	H3	H4	H5	H6	H7	H8	H9
G1	<1e‐5	<1e‐4	<0.05	<1e‐31	<1e‐19	<1e‐22	<1e‐15	<1e‐19	<0.001
G2									<0.01
G3		<1e‐17	<1e‐7	<1e‐14	<1e‐15	<1e‐13	<1e‐8	<1e‐8	
G4						<0.05		<0.001	
G5	<0.001			<1e‐15	<1e‐9	<1e‐7	<1e‐7	<1e‐13	<0.001
G6									
G7									
G8		<1e‐7	<0.05	<0.01	<1e‐6		<0.01	<0.05	
G9				<0.05	<0.01				

The *p*‐values not listed in (C) are not significant.

The groups based on differences in cognition are G1, G4, and G5, while those based on differences in vascular disease (VD) are G2, G6, and G7, and those based on differences in Alzheimer's disease (AD) are G3, G8, and G9. We find the presence or the absence of vascular disease does not make a difference in the biomarkers considered (G2, G6, G7). In the presence of disease, the difference in cognition is more pronounced if AD is present (G5) then if VD is present (G4). If the disease status is ignored then for differences in cognition, every hypothesis was significant (G1). The groups based on the presence or absence of AD factors were different for all the hypotheses, except for sex and BA (G3 and G8). HV was different in greater number of groups and with higher significance than BA.

## Discussion

Cognitive loss in aging results from a heterogeneous group of diseases. Biomarkers can be used to separate cognitively impaired patients into more homogeneous groups to reduce the number of subjects needed in a clinical trial and improve the likelihood of success. We have previously developed a clustering classification method, which we called the double‐dichotomy method by plotting an Alzheimer's disease score on one axis and a vascular disease score on the other.^20^ We made two modifications in this report: (1) we used the Alzheimer's disease neuroimaging initiative (ADNI) database to increase the number of participants, and (2) we added cognitive scores to identify those with and without impairment. This potentially improved subject homogeneity, with each group having well‐characterized biological markers.

Adding the third dimension of cognitive status to the plots created the “trichotomy method” with the benefit of clarifying participants in the MX (bMX) and the preclinical groups (bCN_VD_, bCN_AD_, and bCN_MX_). Diagnosing these patients during life can be done by measuring amyloid beta (Aβ) and phosphorylated tau (pTau) in the CSF or with PET to form an Alzheimer's disease factor (ADF) and using the diffusion MRI scan to indicate vascular damage to form a vascular disease factor (VDF). Neither the ADF nor the VDF directly provide an indication of cognition, leaving out a critical factor for the classification of patients.

Cognitive status and vascular injury are not explicitly considered in the ATN framework. The trichotomy method defines the preclinical groups more clearly, as they have normal cognition, and presence either of AD and/or of VD factors. The bCN_VD_ group is only 3.9% of the ADNI dataset and may represent preclinical vascular cognitive impairment. Similarly, there is the bCN_AD_ group with normal cognition, low VDF, and elevated ADF (13.6%), which would be considered preclinical AD or Stage 1 or 2 disease according to current guidelines.^29^ The MX group (bMX) is more clearly defined as abnormal cognition with elevated ADF and VDF (9.3% in ADNI). The percentage of bMX subjects in the ADNI cohort are lower than the 25% of the subjects in the UNM cohort,^20^ because the ADNI's exclusion criteria reduced recruitment of subjects with significant vascular disease while the UNM cohort involved an enriched number of vascular patients.

It is rare to find a single pathologic process in the brains of demented patients with more than two being the norm and as high as four different pathological processes not uncommon.^30^ This could explain the high failure rate in clinical trials where only one pathological process was treated. This would argue for using a more precise classification process based on biomarkers derived from the underlying brain pathology.

This biomarker approach has not undergone rigorous testing in longitudinal studies with large groups of patients. While the use of the amyloid and tau proteins is widely accepted, studies with a marker for the N component of the formula ATN are few. A recent study also shows how the selection of a biomarker and cutoff for “N” in ATN results in different estimated prevalence of neurodegeneration.^31^ A stronger argument can be made for the inclusion of a vascular factor “V” in the ATN formula because of the ability of MRI to indicate injury to the white matter, which is a strong indicator of vascular injury. However, the optimal set of biomarkers to determine white matter damage remains to be determined.

The large number of subjects with all the data necessary to perform the trichotomy analysis being available in the ADNI database is a strength of the study. Another strength is the proposed method for calculating the normalized composite scores for easier comparison among subjects.

There are several limitations of the study in addition to the lack of longitudinal data. ADNI lacks diversity being mainly composed of white, non‐Hispanic and college educated group of participants and the cutoff values are mainly appropriate for that population. Another limitation is the selection of the variables to include in the vascular disease factor. Inclusion of other vascular measures, such as lacunar infarcts, perivascular space enlargement, and microbleeds would have provided additional validity to the VDF. Recent work has reviewed the role of perivascular spaces in AD,^32^ and elucidated its contribution to early cognitive decline based on the ADNI data.^33^ The methods for automatic quantification of PVS are being developed by different groups, but these methods need to be evaluated for consistency.^34^, ^35^, ^36^ Similarly, there are other factors that could have been included in the cognitive factor.

While the trichotomy separates MX patients from those with AD by including quantitative MRI data, it leaves unresolved the significance of the bCN_VD_ group; the presence of white matter hyperintensities on FLAIR in elderly may be a consequence of aging. Finally, recent technological advances have enabled detection of Aβ42, Aβ40, Tau isoforms, neurofilament light (NfL), and glial fibrillary acidic protein (GFAP) from blood samples and it may be possible to combine these blood‐based markers with MRI white matter markers to make the classification more available for trichotomy classification.^37^, ^38^, ^39^ Ultimately the goal of this research is to provide biomarkers that can be used in a clinical setting. We have not intended for this method to be readily translated into a clinical procedure. However, the MRI biomarkers selected can be obtained from a clinical 3T MRI that is available at most medical centers. Therefore, extracting the PSMD and mFW should be possible. Further work will have to be done to obtain cut‐points for the three axes, and use of other datasets will be needed to bring this into routine clinical work. Our study provides a roadmap for other centers with large datasets and statistical support. The fluid biomarkers were obtained from the CSF, which is not optimal. Although the current state of fluid biomarker research is showing that the plasma‐based biomarkers are undergoing validation against ones from CSF and PET. This will make more widespread the determination of the AD axis and with neuropsychological testing and MRI, the other two axes could be calculated. We expect that in the not‐too‐distant future this type of an approach to classification will become available for clinical research studies and the emerging treatment trials that are based on biomarkers. This approach will be useful with other dementia causing diseases, such as Lewy body disease, limbic‐predominant age‐related TDP43 encephalopathy (LATE), aging‐related tau astrogliopathy (ARTAG), or argyrophilic grain disease (AGD) when appropriate plasma‐based biomarkers are available for them. An improved method of classification has the potential of reducing the numbers of patients needed for a clinical trial. The other advantage of this approach is that it can be used to include patients in rural settings at a distance from the research medical centers.^40^ In summary, we propose that the trichotomy framework can be applied to plasma‐based biomarkers and clinical MRI, making it useful for population‐based studies.

An added advantage is the ability to identify MX during life with multimodal biomarkers and to show different rates of progression of neurodegenerative and vascular disease pathways, providing a guide to which pathway to initiate treatment and when to begin treatment of the second pathway. In this way biomarkers with statistical clustering methods will be the gateway to precision medicine in dementia.

## Author Contributions

The data analysis was done by Caprihan, Hillmer, and Erhardt. The clinical and the neuropsychological data from the University of New Mexico was collected by Adair, Knoefel, Prestopnik, and Rosenberg. The paper was written by Caprihan and Rosenberg and reviewed by all.

## Conflict of Interest

Authors Caprihan, Hillmer, Erhardt, Adair, Knoefel, Prestopnik, and Rosenberg have no conflict of interest and they have no financial interest to disclose related to this work.

## Supporting information


Data S1

## Data Availability

The MRI images and all the data used in this analysis are directly available from the ADNI database. A spreadsheet of the summary measures used in this paper and the python program for statistical analysis and visualization will be available on request to the corresponding author.
