# Supplementary material for: A trichotomy method for defining homogeneous subgroups in a dementia population
Source: Ann Clin Transl Neurol. 2023 Aug 21;10(10):1802–15. doi: 10.1002/acn3.51869 (PMC10578887; doi:10.1002/acn3.51869)
Supplement: Supplementary file 1 — Data S1 [file ACN3-10-1802-s001.docx]

Supplementary Material

The details of the method for calculating the raw and normalized composite scores and the resulting distribution of the subjects across the eight biologically defined groups are described.

1. Calculation of raw vascular disease factor ($VDF_{raw}$)

An independent data set from 94 subjects and with the same biomarkers as ADNI was available from University of New Mexico (UNM) with two well characterized groups of controls and SIVD. The $VDF_{raw}$ is then the distance of the biomarkers from the boundary separating the two groups. The selected biomarkers were white matter mean free water (mFW) and PSMD from diffusion images. Linear discriminant analysis (LDA) (Figure 1A) defined the separating boundary. A robust mean for each of the two groups, and a robust covariance function were calculated based on the minimum covariance determinant (MCD)[1, 2] method (pyod/python package). The sign of the distance was adjusted for positive distances to correspond to increased white matter damage.

1. Calculation of raw cognition score ($Cog_{raw}$)

The raw composite cognition score was based on composite cognition measures ADNI_MEM and ADNI_EF. The ADNI data (538 subjects) was used to define a linear discriminant function that best separated subjects with normal cognition (aCN) and those with some cognitive impairment (aMCI + aAD). An independent data set was not available in this case for calculating $Cog_{raw}$. The previous LDA method was used, and additionally we validated the stability of the $Cog_{raw}$by leave-one-out cross-validation method (Figure 1B).

1. Calculation of raw Alzheimer’s disease factor ($ADF_{raw}$)

The $ADF_{raw}$ was based on the results of an independent study, which used the ratio of Aβ42 to pTau and calculated a cut-off that best matched the PET results for AD presence [3] (Figure 1C).

1. Calculation of UNT

The probability density function of $CS_{raw}$ will typically be different above and below the cut-off $cth$, because in most databases the distribution of controls is different from that of subjects with some impairment (Figures 1D, 1E, and 1F). We separately calculate the probability density function $f_{H}(CS_{raw})=f(CS_{raw}/CS_{raw}>cth)$ and $f_{L}(CS_{raw})=f(CS_{raw}/CS_{raw}\leq cth)$ for above (H) and below (L) the cut-off threshold. We then use their probability distribution functions to map $f_{H}(CS_{raw})$ from the range $[cth,\infty]$ to the range [0.5, 1] and map $f_{L}(CS_{raw})$ from the range $[-\infty,cth]$ to the range [0.0, 0.5]. The normalized composite score, $CS_{norm}$ is given by,

$CS_{norm}=\left\{\begin{matrix} 0.5(1+\int_{cth}^{CS_{raw}} f_{H}(u)du)\text{ , for }CS_{raw}>cth \\ 0.5(1-\int_{CS_{raw}}^{cth} f_{L}(u)du)\text{, for }CS_{raw}\leq cth \end{matrix}\right.$ . [1]

The probability density function of $CS_{norm}$ is theoretically uniform in the interval [0,1]. In practice, because of a finite sample-size it is only approximately uniform.

Each raw composite score was transformed to a normalized composite score by implementing Eq. [1] as a table (Figures 1G, 1H, and 1I).

1. Trichotomy-based subgroups

Table S1: Distribution of ADNI subjects across the eight biologically defined sub-groups is given.

| ADNI Biological Subgroups | Cognitively  Normal  aCN | Mild Cognitive Impairment  aMCI | Alzheimer’s Disease  aAD | Number of Subjects |
| --- | --- | --- | --- | --- |
| bMX | 7 | 21 | 22 | 50 (9.29%) |
| bAD | 7 | 66 | 42 | 115 (21.38%) |
| bVD | 5 | 9 | 0 | 14 (2.60%) |
| bCL | 19 | 33 | 4 | 56 (10.41%) |
| bCN_MX_ | 10 | 3 | 1 | 14 (2.60%) |
| bCN_AD_ | 56 | 17 | 0 | 73 (13.57%) |
| bCN_VD_ | 17 | 4 | 0 | 21 (3.90%) |
| bCN | 162 | 33 | 0 | 195 (36.25%) |
| Total | 283 | 186 | 69 | 538 |


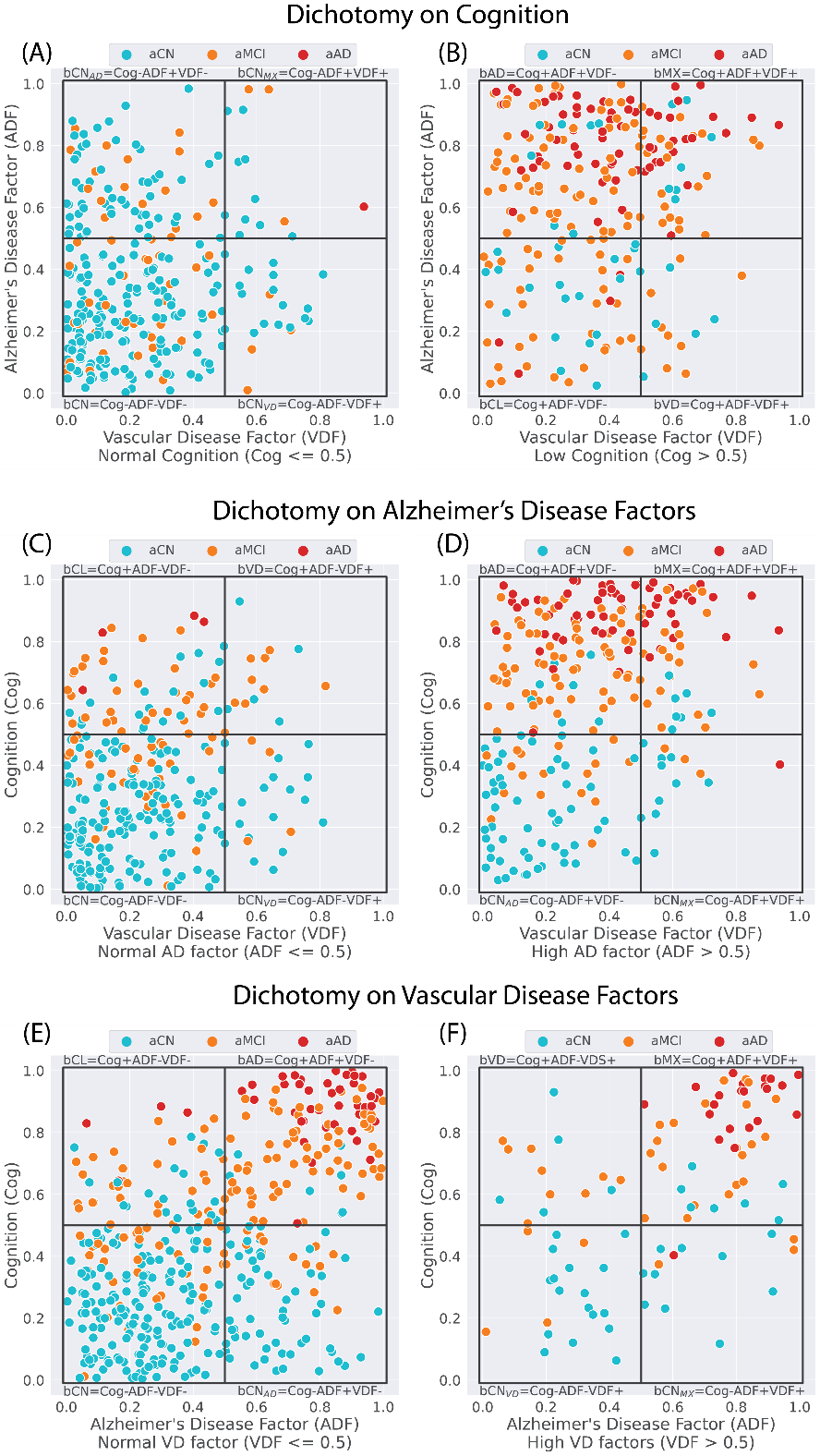


Figure S1. The trichotomy plot in three dimensions (VDF, ADF, Cog) has been divided into six figures, based on a different variable’s dichotomy for each row. The colors distinguish the original ADNI classification (aCN, aMCI, and aAD). This figure reflects the characteristics of the ADNI database. Majority of the subjects fall in bCN and bAD groups (57.6%, Fig. 3E) with considerably lower number of subjects have the vascular disease factor (18.4%, Fig. 3F). This analysis identifies subjects with mixed dementia (bMX) and those considered to have preclinical vascular disease (bCN_VD_).

References

[1] Rousseeuw PJ, Van Driessen K. A fast algorithm for the minimum covariance determinant estimator. Technometrics. 1999;41:212-23.

[2] Hubert M, Debruyne M, Rousseeuw PJ. Minimum covariance determinant and extensions. Wires Comput Stat. 2018;10.

[3] Hansson O, Seibyl J, Stomrud E, Zetterberg H, Trojanowski JQ, Bittner T, et al. CSF biomarkers of Alzheimer's disease concord with amyloid-beta PET and predict clinical progression: A study of fully automated immunoassays in BioFINDER and ADNI cohorts. Alzheimers Dement. 2018;14:1470-81.
